# Information Bottleneck Classification in Extremely Distributed Systems

**DOI:** 10.3390/e22111237

**Published:** 2020-10-30

**Authors:** Denis Ullmann, Shideh Rezaeifar, Olga Taran, Taras Holotyak, Brandon Panos, Slava Voloshynovskiy

**Affiliations:** SIP—Stochastic Information Processing Group, Computer Science Department CUI, University of Geneva, Route de Drize 7, 1227 Carouge, Switzerland; denis.ullmann@unige.ch (D.U.); shideh.rezaeifar@unige.ch (S.R.); olga.taran@unige.ch (O.T.); taras.holotyak@unige.ch (T.H.); brandon-leigh.panos@etu.unige.ch (B.P.)

**Keywords:** information bottleneck principle, classification, deep networks, decentralized model, rate-distortion theory

## Abstract

We present a new decentralized classification system based on a distributed architecture. This system consists of distributed nodes, each possessing their own datasets and computing modules, along with a centralized server, which provides probes to classification and aggregates the responses of nodes for a final decision. Each node, with access to its own training dataset of a given class, is trained based on an auto-encoder system consisting of a fixed *data-independent encoder,* a pre-trained *quantizer* and a *class-dependent decoder.* Hence, these auto-encoders are highly dependent on the class probability distribution for which the reconstruction distortion is minimized. Alternatively, when an encoding–quantizing–decoding node observes data from different distributions, unseen at training, there is a mismatch, and such a decoding is not optimal, leading to a significant increase of the reconstruction distortion. The final classification is performed at the centralized classifier that votes for the class with the minimum reconstruction distortion. In addition to the system applicability for applications facing big-data communication problems and or requiring private classification, the above distributed scheme creates a theoretical bridge to the information bottleneck principle. The proposed system demonstrates a very promising performance on basic datasets such as MNIST and FasionMNIST.

## 1. Introduction

**Figure 1:**
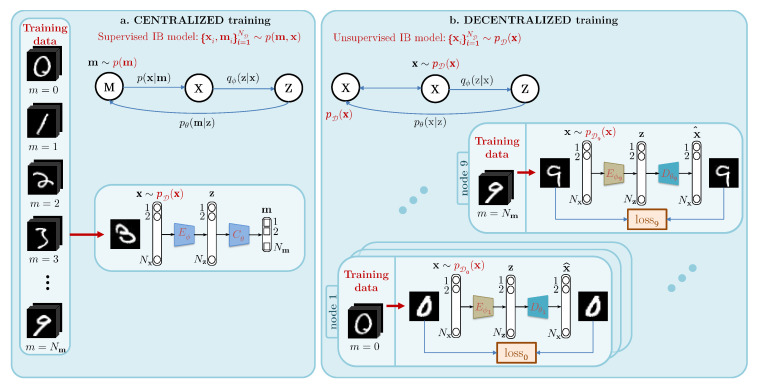
Theoretical and practical differences between centralized and decentralized training: (**a**) for the centralized training, the model can access all available data and, therefore, learn decision boundaries between classes. It is usually a single supervised classifier. More generally, it can be decomposed into an encoder followed by a classifier: the data manifold is projected by *E* onto a constrained space to make the work of *C* simpler as in [5]. In theoretical terms, this model is justified by the Information Bottleneck (IB) principle [6] described by the Markov chain above and corresponds to the IB for supervised models described in [7]; (**b**) for the fully decentralized training, we assume the scenario, where each node has an access to the training data of one class only. The model cannot learn the decision boundaries between classes contrary to the centralized one. Each node is following the unsupervised IB model described in [7]. They share the same *E* and *D* structure but the parameters of encoders $\phi_{0},\cdots ,\phi_{N_{m}}$, and decoders $\theta_{0},\cdots ,\theta_{N_{m}}$ are learned for each class individually, given by the data manifold of each class. At the classification stage, the nodes share only the reconstruction errors with the central node.

Most classical machine learning architectures are based on a common classifier that typically requires centralizing all the training data in a common data center for training as schematically shown in Figure 1a. However, such a centralized system faces several critical requirements related to data privacy and the need for big-data communications to collect data of all classes at the central location. In practice, sensitive data such as medical and financial records or any personal data are usually kept private in multiple independent data centers and cannot be shared between third parties for various reasons. At the same time, a huge amount of newly acquired private data that requires special care when fed to a machine learning tool, is captured daily. However, from both privacy and practical points of view, it is not feasible to transfer all collected data to a centralized data center and to perform the system re-training on new data. To face these challenges, a concept of “Decentralized machine learning” is proposed and developed in several works, where the data are stored locally on the devices and a common centralized model is trained. Not pretending to be exhaustive in our overview, we mention some of the most important contributions within the literature. “Parallelized SGD” was introduced in 2007 [1] and further extended in [2] to reduce the communication costs using compression or pruning algorithms. An alternative solution known as “Federated Averaging” was proposed in [3] with many attempts to improve the performance and communication cost as in [4].

The term *Federated Learning* (FL) is used for a type of decentralized learning, where a global model is kept in a central node/device and many local nodes/devices have different amounts of samples from different classes. In FL, the local and/or global nodes share the gradients or model parameters during training by efficient techniques such that *RingAllReduce* [8] for gradients sharing or *Federated Averaging* [4] for local model parameters averaging on the central node, and *Ensemble Learning* [9] for local predictions averaging. When all devices have samples from all classes in equal amounts, the setup is commonly referred to as *Independent Identically Distributed Federated Learning* (IID-FL). However, in practice, it is often that different nodes/edges/devices might have samples only from some classes in different proportions. Such an unconstrained environment would almost always mean that not all edge devices will have data from all the classes. This is commonly referred to as a *Non-Independent Identically Distributed Federated Learning* (Non-IID-FL). This represents a real challenge for FL and leads to significant drops in classification accuracy. Recently, many works propose solutions to cope with this problem, such as mixing Federated Averaging with Ensemble Learning [10], incorporating recent communication and data-privacy amplification techniques [11], sharing small subsets of IID training data among the local nodes [12], adapting the local nodes communication frequencies to the skewness [13], and efficiently defending communications between nodes [14]. In [13], the authors compare the performance of different classification architectures on the IID and Non-IID data with different training tricks and demonstrate a significant drop in performance for Non-IID data. Therefore, to the best of our knowledge there is a significant gap between the performance of centralized systems and the Non-IID-FL systems.

**The challenges of decentralized classification.** There is a gap between the classification results of centralized and decentralized classification, even for simple datasets such as MNIST [15], for which a very high recognition accuracy was achieved on centralized models while the performance in the decentralized setting is quite modest. Studies such as [16,17] showed that semi-supervised classification is even a more challenging task for such systems. Anomaly detection and one-class classification are two subfamilies of unsupervised learning closely related to decentralized classification. Well-known methods such as one-class support vector machines [18] are proposed, but in practice they suffer from very slow training and limited performance. To the best of our knowledge, all recent advances in anomaly detection use generative models composed of encoding and decoding, with adversarially learned denoising to better separate in- and outlier images [19], or by adversarially learning a disentangled implicit representation [20], or by constraining a latent representation to generate only possible examples from the class and to avoid generating any example outside the class, no matter how close it is to the class [21]. These studies state without theoretical explanation that their models seek to project and compress the data distribution of a class in an optimal way to keep only the information necessary to identify the class in question while being able to regenerate the initial data from the compressed version with minimal error. However, the most recent studies clearly demonstrate the critical limitations of trained representations on single class data. As shown in [21], if the model is not sharpened to reject outlier samples, it can learn more generic information than is strictly necessary for a given class, in which case it is unable to isolate that class from the others. This is, for instance, the case in [21], where the one-class model was trained on class 8 and yet considers outlier classes 1, 5, 6 and 9 as inlier class 8.

## 2. Problem Formulation: The One Node–One Class Setup

**Figure 2:**
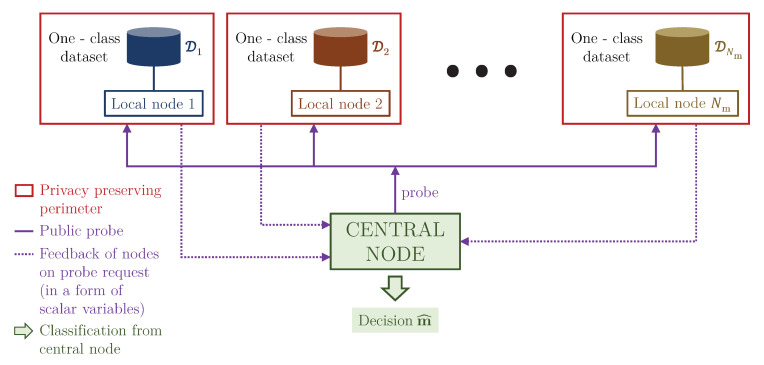
Classification setup under the analysis in this paper. It shows which parameters of the system are under the privacy protection and which are shared in the public domain. Also, the hyperparameters such as the learning rate and the number of epochs are in the public domain, sent from the central node to all local nodes.

Contrary to the centralised classification presented in Figure 1a, each class is assigned to one decentralized training node, which learns to optimally compress and decompress in-class data as shown in Figure 1b. The setup under analysis of this paper is shown in Figure 2. We assume that the system consists of $N_{m}$ local nodes and one centralized node. Each node has access to its own privacy sensitive dataset. The entries of each local dataset are generated from one-class distribution. The centralized node does not have any access to the local node datasets and cannot receive any information about the updates of gradients typically considered in the FL settings. The only information that can be exchanged between the local nodes and centralized node is the probe, which is considered to be a public one, and the feedback of local nodes in a form of scalar variables. Therefore, the communication between the local nodes and centralized node is reduced to minimum at the testing stage. At the training stage, we assume no communication between the centralized node and local nodes. Additionally, the local nodes do not share any information between them. Up to our best knowledge such a scenario was not addressed in known FL systems.

The privacy protection model considered in this work has an asymmetric character. We only address the privacy protection of owner datasets, i.e., the training data. At the same time, the probe to be classified at the testing stage is not considered to be privacy sensitive one. Therefore, we assume that it can be shared in the plain form among different nodes. Although our model can also address the privacy protection mechanism for the probe, this problem is out of scope of this paper. We also assume that the nodes are playing a fair game and do not modify or taper their feedback to the centralized node. Therefore, the model under investigation assumes the following setting.

*At the training stage* we assume an extreme case of a Non-IID system setup, where each node/device has an access only to the samples of a single class/distribution. According to this assumption we want to address many practical scenarios, where the nodes representing some institutions like labs and research centers, companies, service providers, individuals or even countries, do not want or cannot share their data with each other for various reasons that include for example privacy concerns, competition issues, national security interests, etc. as well as technical constrained related to the transfer of big volumes of data via band-limited channels in a restricted amount of time. At the same time, the data owners representing such kind of nodes are interested in providing classification services to some third parties based on the possessed data without revealing it explicitly to any third party. There are numerous examples of considered scenarios ranging from privacy sensitivity medical records or biological research, where some particular institutions, which are specialized on study of some disease or phenomena, invest considerable amount of time, efforts and money to collect such kind of data. In addition, one institution might possess data from healthy population and yet the others with some specific diseases. Obviously, these institutions would be interested in sharing their data to reveal new discoveries but cannot proceed due to the above economical, privacy or competition reasons. One can also envision a scenario of personalized marketing, where each node represents a client or a company that has some unique experience or interest expressed by the data collected from its activity in certain domain. The advertising party suggests some services or product to all clients by sending a probe and if there is a match between the interest and proposal, a deal is concluded. At the same time, it is obvious that the interests of each client are private. The scenarios of astronomical or genetic research might also face big-data communication concerns, where a lot of data are collected and labeled at some distributed locations and to transfer all these data to a central node might represent a great technical or economic challenge. Additionally, the situation might be complicated by a need for regular data updates. All these scenarios are evidently exemplified on systems like Square Kilometer array (SKA) [22], where the data are planned to be collected on two continents with a rate of 1 Petabyte per day, with the envisioned daily transfer to a centralized location by an airplane.

Therefore, in the considered setup we assume very restricted communications between local and central nodes. Furthermore, we assume that no global model is stored in the central node and the nodes have no communications with the central node in terms of both sharing samples (local class in- or outliers) and gradients or parameters in the open or obfuscated form.

*At the testing stage* we address the classification problem. We assume that the central node has a probe that is not private, and it can be openly communicated between the nodes. In this way, the privacy of the probe is not considered in our work.

During the classification, the local nodes only communicate the reconstruction error in the form of a scalar to the central node, thus allowing for efficient and fast training and classification even when the local nodes are distributed around the world. For instance, this can be the case for astronomy observation centers which contain large quantities of data, where the training and classification have high transmission restrictions. Such a problem formulation is not directly addressed in the FL formulation and to the best of our knowledge there are no results reporting the performance of FL on this extreme Non-IID setup. We refer to this particular case of Non-IID data as *One Node–One Class* (ON-OC) setup.

**Figure 3:**
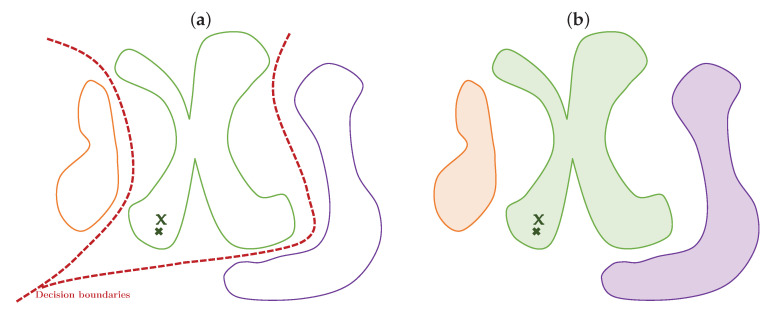
Conceptual difference between (**a**) the centralized classifications and (**b**) the extremely decentralized ON-OC classification. Colors represent the manifolds of each learned class. (**a**) Centralized and Federated classification; (**b**) ON-OC classification.

The considered classification setup has a significant conceptual difference with the centralized classification systems. Centralized classification is based on the notion of a decision boundary between the classes that should be learned by observing multiple training samples from all classes simultaneously, as shown in Figure 3a. The classification is based on a decision to which region of space, split by the decision regions, a probe x belongs to. In the fully distributed case, referred to as the ON-OC setups, where no gradient is shared between nodes, the proposed system learns manifolds of each data class independently represented by colors in Figure 3b. The encoding-decoding of x achieves this by class-dependent encoder–decoder systems producing minimum reconstruction error for the matched probe case.

**Figure 4:**
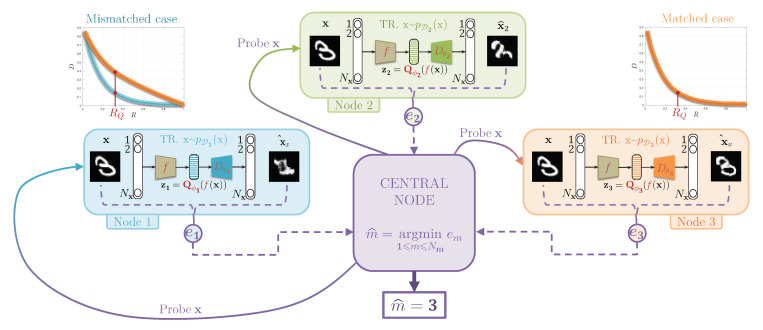
At testing time, the probe x is sent from the central node to $N_{m}$ compression-decompression local nodes, trained on their own data (“TR.” denotes “trained”), e.g., $N_{m}=3$ for this example. The results of decompression expressed in the reconstruction error denoted as $e_{1}$, $e_{2}$ and $e_{3}$ are sent back to the central node. The proposed distributed model classifies in favor of the smallest reconstruction error. The compression in each node is characterized by a compression rate $R_{Q}$, which is chosen to be such that the distortion distributions for mismatched classes are maximized with respect to the matched case.

We propose a theoretical justification and proof of concept for a fully decentralized classification model, where the classifier training procedure is not required to see all class data at the same time to achieve a high accuracy classification. More precisely, we assume that each class is assigned to one decentralized training node, which learns to optimally compress and decompress in-class data, such that the reconstruction error is minimized for in-class data, and the latent compressed representation learns in-class data manifold instead of inter-class boundaries (Figure 3). At the same time, the presented framework can be extended to the more general case of multiple classes per node. In this case, the nodes can benefit from a priori simpler semi-supervised training, or at least they can train as many models as the number of classes per node, given that they have enough data. Once the training is completed, the classification step presented in Figure 4 is as follows: the central node sends a sample x, a *probe*, from the data distribution to be classified, to each of the local nodes. These local nodes are optimized on a single class to compress and decompress and only the reconstruction errors of the probe are transmitted from each node to the central node, which votes in favor of a class with the lowest error.

## 3. Related Work

In contrast to the Federated Learning-based classification considered in Section 1, in this section we will consider concepts related to the proposed framework.

**An information bottleneck interpretation.** We use the Information Bottleneck (IB) principle presented in [6] to build the theory behind centralized and decentralized classification models. The analysis of the supervised and unsupervised information bottleneck problems was performed in [23] and generalized to the distributed setup in [24]. In this work, we extend the IBN to demonstrate the importance of compression in the form of vector quantization for the classification problem. Moreover, we show that the classical centralized training is a *supervised IB* scenario whereas the decentralized one is an instance of an *unsupervised IB* scenario as developed in [7] and summarized in Figure 1. Ideally, each node should: (a) store in its encoded parameters the in-class data information to ensure the distribution of one class to be distinct from the other ones, (b) be trained to compress and decompress optimally for in-class data, such that the reconstruction error is minimized (blue rate-distortion curve in Figure 4 of the matched case), and sub-optimally for out-of-class data, such that the reconstruction error is not minimum (orange rate-distortion curve of the same mismatched case), and (c) have a rate of compression ($R_{Q}$ in Figure 4), which separates the optimal node from sub-optimal ones. Shannon’s rate-distortion theory assumes that the compression-decompression model used for the data compression should be jointly trained for input data statistics. This makes a link to optimal matched signal detection used in the theory of signal processing: each class has its own representative manifold and a corresponding *filter* represented by its proper encoder–decoder pair. The main difference with the matched filter, is that this filter is designed for one particular signal. Thus, the matched filter detects the closeness of the probe to the signal. In our framework, we validate the proximity of the signal to the entire class manifold represented by the ensemble of training data. However, it is not done by measuring the proximity of each available training in-class data point and aggregating the results, but instead by the trained model itself, ensuring a *continuity* of the learned data manifold that is achieved by the considered encoder–decoder system as whole. It is important to note that compression is not required for such learning. Instead, the compression is needed to distinguish in-class and out-class probes by providing higher reconstruction error for the out-class samples, as shown in Figure 4 for the mismatched case plot.

**Big-data and privacy-preserving classification.** In the considered setup, the notion of privacy concerns the training data sets that are kept locally in each node. No data sharing or model parameter sharing is required either between the local nodes or centralized server. Therefore, the training stage is considered to be privacy-preserving one. At the same time, we assume that the probe distributed by the centralized node for the classification is not considered to be privacy sensitive one at the classification stage. Therefore, no special measures are taken to preserve its privacy. At the same time, one can assume special obfuscation strategies for the probe protection like randomization of special dimensions in the embedded space and we refer an interested reader for the overview of such techniques in [25].

The potential benefits of the considered architecture are as follows: (a) there is no need to transfer all of the data or gradients to a centralized location (large-scale applications); (b) data privacy is ensured by keeping data and model parameters locally; (c) the reconstruction score is produced locally and also (d) it might eliminate a potential vulnerability against adversarial attacks by preventing the ability to learn a sensitive classification boundary. To fully benefit from these attractive features, we have to validate the performance of the proposed distributed classification architecture against the classical fully supervised architecture that has access to all training samples simultaneously for the optimal decision rule. This is the targeted goal addressed in the current paper.

**Novelty and contribution:**

1. We propose a fully distributed learning framework without any gradient communication to the centralized node as it is done in the distributed systems based on FL. As pointed out in [11,26] this resolves many common issues of FL related to the communication burden at the training stage and the need for gradient obfuscation for privacy reasons.
2. We consider a new problem formulation of decentralized learning, where each node has an access only to the samples of some class. No communication between the nodes is assumed. We call this extreme case of Non-IID Federated Learning as ON-OC setup.
3. We propose a theoretical model behind the proposed decentralized system based on the information bottleneck principle and justify the role of lossy feature compression as an important part of the information bottleneck implementation for the considered ON-OC classification.
4. In contrast to the centralized classification systems and distributed Federated Learning, which both mimic the learning of decision boundaries between classes based on the simultaneously available training samples from all classes, we propose a novel approach, which tries to learn the data manifolds of each individual class at the local nodes and make the decision based on the proximity of a probe to each data manifold at the centralized node.
5. The manifold learning is also accomplished in a new way using a system similar to an auto-encoder architecture [27] but keeping the encoder fixed for all classes. Thus, the only learnable parts of each node are compressor and decoder. This leads to the reduced training complexity and flexibility in the design of compression strategies. Additionally, by choosing the encoder based on the geometrically invariant network a.k.a. *ScatNet* [28], one can hope that the amount of training data needed to cope with the geometrical variability in training data might be reduced as suggested by the authors of [28].
6. Finally, the proposed approach also differs to our previous framework [29] in the following way:
  - The framework in [29] was not based on the IB principle, while the current work explicitly extends the IB framework.
  - The previous work [29] did not use the compression in the latent space while the current work uses an explicit compression in a form of a vector quantization. The use of quantization is an important element of the IB framework in the considered ON-OC setup. In this work that the results of classification with the properly selected compression are considerably improved with respect to the unquantized latent space case considered in our prior work [29].
  - The [29] was based on the concept of Variational Auto-Encoder (VAE), which includes the training of the encoder and decoder parts. This requires sufficient amount of data to obtain the invariance of the encoder to the different types of geometrical deviations. At the same time, the current work is based on the use of geometrically invariant transform, in particular ScatNet, which is designed to be invariant to the geometrical deviations. This allows, first of all, to avoid the training of encoder and, secondly, to train the system without big amount of labeled data or necessity to observe the data from all classes.
  - In the case of VAE-based system the latent space is difficult to interpret in terms of the selection of dimensions for the quantization. In the case of use of ScanNet as an encoder part the latent space is well interpretable, and its different sub-bands correspond to different frequencies. In this respect, it becomes evident which sub-bands should be preserved and which ones could be suppressed (depending on the solved problem).
  - Finally, this new setup shows higher classification accuracy for the ON-OC setup.

## 4. Theoretical Model

### 4.1. Information Bottleneck Concept of Centralized Systems

The theory of the centralized classification model is based on the recently proposed *Information Bottleneck* (IB) principle [6]. In a centralized classification model, the training samples are taken from the available labeled data of all $N_{m}$ classes: ${\left\{x_{i},m_{i}\right\}}_{i=1}^{N_{D}}∼p_{D}\left(m,x\right)$, where $N_{D}$ corresponds to the number of training samples. It corresponds to the supervised version [7] of the IB with a variational approximation, where the model learns to minimize the mutual information $I_{\phi }\left(X;Z\right)$ between the labeled data X and the latent representation Z, while retaining the mutual information $I_{\phi ,\theta }\left(Z;M\right)$ between the latent representation Z and class label M larger than some value $I_{m}$. This explains a compression of X by means of a parametrized encoding $q_{\phi }\left(z|x\right)$ such that Z is a sufficient statistics for M, allowing the training of a mapper to classify from Z to M, using $p_{\theta }\left(m|z\right)$. Figure 1a describes the architecture of transmission of information $M\overset{p(x|m)}{\to }X\overset{q_{\phi }\left(z|x\right)}{\to }Z\overset{p_{\theta }\left(m|z\right)}{\to }M$. The parameters ϕ for compressing X into the latent representation Z, and θ for classifying Z into M, are jointly trained to optimize the Lagrangian of the supervised IB developed in [7] as:

(1) $$\left(\hat{\phi },\hat{\theta }\right)=\underset{\left(\phi ,\theta \right)}{arg min}L^{S}\left(\phi ,\theta \right),\;\mathrm{with}\;L^{S}\left(\phi ,\theta \right)=I_{\phi }\left(X;Z\right)-\beta I_{\phi ,\theta }\left(Z;M\right),$$

where S stands for the supervised setup and β is a regularization parameter corresponding to $I_{m}$. Moreover, the mutual information between the input Z and the output M of the classification can be decomposed as:

(2) $$I_{\phi ,\theta }\left(Z;M\right)=H\left(M\right)-H_{\phi ,\theta }\left(M|Z\right),$$

where M is a categorical variable whose realizations are one-hot-class encoded vectors m of dimension $N_{m}$ corresponding to the number of classes. As a result, assuming that all classes are equiprobable, the value of H(M) is determined as $H\left(M\right)=\log_{2}\left(N_{m}\right)$, and therefore not parametrized, which leads to:

(3) $$\left(\hat{\phi },\hat{\theta }\right)=\underset{\left(\phi ,\theta \right)}{arg min}H_{\phi }\left(Z\right)-H_{\phi }\left(Z|X\right)+\beta H_{\phi ,\theta }\left(M|Z\right),$$

where $I_{\phi }\left(X;Z\right)=H_{\phi }\left(Z\right)-H_{\phi }\left(Z|X\right)$. The common classification models therefore optimize these three terms simultaneously, and we have the following interpretations for Equation (3):

- A minimization of $H_{\phi }\left(Z\right)$ such that Z should contain as little information as possible about X for compression purposes; therefore one has to *compress* at the encoding $X\overset{q_{\phi }\left(z|x\right)}{\to }Z$. In general, this compressing encoding is learned by optimizing ϕ. We simplified the learning process by using a deterministic compression map $Z=Q_{\phi }\left(f_{\phi }\left(X\right)\right)$, where $f_{\phi }\left(\cdot \right)$ is a feature extractor and $Q_{\phi }\left(\cdot \right)$ is a vector quantizer. Accordingly, the rate $R_{Q}=H_{\phi }\left(Z\right)\leq \log_{2}K$ is determined by the number of centroids *K* in the considered vector quantizer, with equality, if and only if all centroids are equiprobable.
- A maximization of $H_{\phi }\left(Z|X\right)$ under the deterministic encoding $Z=Q_{\phi }\left(f_{\phi }\left(X\right)\right)$ reduces to zero and thus: $H_{\phi }\left(Z|X\right)=0$ in Equation (3).
- A minimization of $H_{\phi ,\theta }\left(M|Z\right)$, which represents the cross-entropy between the distribution of the true labels p(m) and the estimated ones $p_{\theta }\left(m|z\right)$:
  (4) $$H_{\phi ,\theta }\left(M|Z\right)=-E_{p(x,m)}\left[E_{q_{\phi }\left(z|x\right)}\left[\log_{2}p_{\theta }\left(m|z\right)\right]\right],$$

with p(x,m)=p(m)p(x|m).

Finally, under the deterministic compressing encoding $Z=Q_{\phi }\left(f_{\phi }\left(X\right)\right)$, we can conclude that the low rate, $R_{Q}$ achievable with smaller *K*, corresponds to higher compression and increased distortion, and as a result increased, $H_{\phi ,\theta }\left(Z|X\right)$ and leads to the minimization of $I_{\phi }\left(X;Z\right)=H_{\phi }\left(Z\right)-H_{\phi }\left(Z|X\right)$ in Equation (1). At the same time, Z should contain enough information about M, which is controlled by the term $\beta H_{\phi ,\theta }\left(M|Z\right)$ in Equation (3) and by the term $\beta I_{\phi ,\theta }\left(Z;M\right)$ in Equation (1). Under the fixed rate $R_{Q}$, one trains the decoder $p_{\theta }\left(m|z\right)$ that simultaneously represents a classifier:

(5) $$\begin{array}{c} \hat{\theta }=\underset{\theta }{arg min}H_{\phi ,\theta }\left(M|Z\right),\;\mathrm{where}\;Z=Q_{\phi }\left(f_{\phi }\left(X\right)\right),\\ \mathrm{then}\;\hat{\theta }=\underset{\theta }{arg max}E_{p(x,m)}\left[\log_{2}p_{\theta }\left(m|Q_{\phi }\left(f_{\phi }\left(x\right)\right)\right)\right]. \end{array}$$

This setup represents many classical state-of-the-art centralized fully supervised classifiers trained based on the maximum likelihood in Equation (5).

### 4.2. Information Bottleneck Concept of Decentralized Systems

In the general case, in contrast to the centralized systems considered above, the proposed decentralized classification is based on the $N_{m}$ nodes, each representing an unsupervised system, and the centralized node that distributes the probes for classification, and collects $N_{m}$ scores for the final decision. Therefore, given a training set ${\left\{x_{i}\right\}}_{i=1}^{N_{D_{m}}}$ for each class $m\in \left\{1,\cdots ,N_{m}\right\}$ generated from $x∼p_{D_{m}}\left(x\right)$ as shown in Figure 1b, each decentralized unsupervised system includes an encoder $E_{\phi_{m}}\left(\cdot \right)=Q_{\phi_{m}}\left(f\left(\cdot \right)\right)$, decomposed in a deterministic data-independent feature extraction f(·) followed by a trainable compression $Q_{\phi_{m}}\left(\cdot \right)$ and a parametrized decoder $D_{\theta_{m}}$.

The training of unsupervised nodes is based on the unsupervised IB considered in [7] (see Figure 1b):

(6) $$\left({\hat{\phi }}_{m},{\hat{\theta }}_{m}\right)=\underset{\left(\phi_{m},\theta_{m}\right)}{arg min}L^{U}\left(\phi_{m},\theta_{m}\right),\;\mathrm{with}\;L^{U}\left(\phi_{m},\theta_{m}\right)=I_{\phi_{m}}\left(X;Z\right)-\beta I_{\phi_{m},\theta_{m}}\left(Z;X\right),$$

where U stands for the unsupervised setup and similarly to the supervised counterpart:

(7) $$\begin{array}{c} I_{\phi_{m}}\left(X;Z\right)=H_{\phi_{m}}\left(Z\right)-H_{\phi_{m}}\left(Z|X\right)=H_{\phi_{m}}\left(Z\right)=\log_{2}\left(K\right),\\ \mathrm{and}\;I_{\phi_{m},\theta_{m}}\left(Z;X\right)=H_{\phi_{m},\theta_{m}}\left(X\right)-H_{\phi_{m},\theta_{m}}\left(X|Z\right). \end{array}$$

In this work, we will assume that $H_{\phi_{m},\theta_{m}}\left(X\right)=H_{D}\left(X\right)$ is independent of encoding-decoding parameters and represents the entropy of the training dataset and:

(8) $$H_{\phi_{m},\theta_{m}}\left(X|Z\right)=-E_{p_{D}\left(x\right)}\left[E_{q_{\phi_{m}}\left(z|x\right)}\left[\log_{2}p_{\theta_{m}}\left(x|z\right)\right]\right],$$

represents the conditional entropy that is determined by the decoder $p_{\theta_{m}}\left(x|z\right)$. Assuming that $p_{\theta_{m}}\left(x|z\right)\propto e^{-d(x,D_{\theta_{m}}\left(z\right))}$, one can interpret $\log_{2}p_{\theta_{m}}\left(x|z\right)\propto -d\left(x,D_{\theta_{m}}\left(z\right)\right)$, where $d(x,D_{\theta_{m}}\left(z\right))$ denotes the distortion function between x and its reconstructed counterpart $\hat{x}=D_{\theta_{m}}\left(z\right)$. Accordingly, for the considered non-stochastic encoding $Z=Q_{\phi_{m}}\left(f\left(X\right)\right)$, Equation (8) reduces to $H_{\phi_{m},\theta_{m}}\left(X|Z\right)=-E_{p_{D}\left(x\right)}\left[d(x,D_{\theta_{m}}\left(Q_{\phi_{m}}\left(f\left(x\right)\right)\right))\right]$ and:

(9) $${\hat{\theta }}_{m}=\underset{\theta_{m}}{arg min}E_{p_{D}\left(x\right)}\left[d(x,D_{\theta_{m}}\left(Q_{\phi_{m}}\left(f\left(x\right)\right)\right))\right].$$

**Figure 5:**
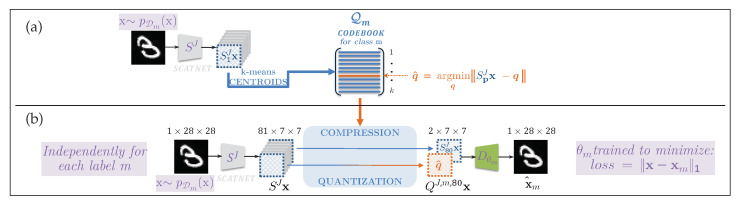
A detailed architecture of proposed model for a local node compression: (**a**) the generation of a dictionary to quantize a portion of the scattering transform feature vector, (**b**) the processing chain for encoding, compression and regeneration.

The encoder in the considered setup consists of data-independent transform f(.) and trainable quantizer $Q_{\phi_{m}}\left(\cdot \right)$. There are several ways to implement such a quantizer. In this paper, we consider a vector quantizer that consists of a codebook $Q_{m}$. Practically, the centroids of this codebook are learned using K-means algorithm and the quantization procedure consists of searching the closest centroid in the codebook to each entry as explained in Figure 5. Each class is represented by its own $K_{m}$ centroids.

Therefore, given a training set ${\left\{x_{i}\right\}}_{i=1}^{N_{D_{m}}}$ for each class $m\in \left\{1,\cdots ,N_{m}\right\}$ generated from $x∼p_{D_{m}}\left(x\right)$ as shown in Figure 1b, each node trains its own encoder–decoder pair $\left(E_{\phi_{m}},D_{\theta_{m}}\right)$, i.e., the compressor $Q_{\phi_{m}}\left(\cdot \right)$ and the parameters of the decoder $\theta_{m}$ according to:

(10) $$L^{U}\left(\phi_{m},\theta_{m}\right)=\log_{2}K_{m}+\beta \sum_{i=1}^{N_{D_{m}}}d(x_{i},D_{\theta_{m}}\left(E_{\phi_{m}}\left(x_{i}\right)\right),$$

where $K_{m}$ is several centroids for each class. The total number of centroids for all classes is bounded that corresponds to the constrain on the total allowable rate. One can easily notice that the first term represents the rate of latent space and the second one the reconstruction distortion. Therefore, in this formulation, the unsupervised IB reduces to the rate-distortion formulation [30] averaged over all classes/nodes. This also explains the role of the rate-distortion function shown in Figure 4. For our experiments, the compression ratio is not learned and the structure of the compressing encoder $E_{\phi }\left(\cdot \right)=Q_{\phi }\left(f\left(\cdot \right)\right)$ allows us to set this ratio to be fixed to meet certain requirements considered below. In case of the fixed number of centroids per class considered in this paper, one can skip the term $\log_{2}K_{m}$ in (10).

Once trained, the $N_{m}$ nodes return the distortions $e_{m}=d\left(x,D_{\theta_{m}}\left(Q_{\phi_{m}}\left(f\left(x\right)\right)\right)\right),\;m=1,\cdots ,N_{m}$ for each probe x. The centralized node receives all distortions ${\left\{e_{m}\right\}}_{m=1}^{N_{m}}$ and picks up the minimum one as the result of classification:

(11) $$\hat{m}=\underset{1\leq m\leq N_{m}}{arg min}e_{m}.$$

The detailed architecture of our model for a local node is sketched in Figure 5. The chosen encoding strategies for the scattering feature extractor *f* and the compression $Q_{\phi_{m}}$ are detailed in Section 5.1.

Our architecture resembles the principles of multi-class classification with the independent encoding of each class. Our approach is also linked to the information theory of digital communications. In the classical Shannon’s communication theory, the asymptotic equipartition property ensures that the capacity of the communication system is asymptotically achieved using $N_{m}$ independent binary classifiers known as jointly typical decoders assigned to each message to be communicated [30], Chap. 3. As with these frameworks, we want to build $N_{m}$ distributed classifiers ensuring unified classification, e.g., decoding. However, instead of using $N_{m}$ binary classifiers, we will use a compression framework, which assumes that each class has its own optimal compressor-decompressor pair in terms of reconstruction error. If the probe comes from a corresponding class, its compression-decompression distortion is minimum for a chosen rate $R_{Q}$, while if it is not the case, the distortion is maximized. The compressor-decompressor pair corresponds to an encoder-quantizer-decoder setup, where the latent space vector is quantized to a certain number of bits or rate $R_{Q}=\log_{2}\left(K\right)$. We investigate an extreme case, when the encoder has the same architecture for all classes consisting of the data-independent feature extractor and the quantizer that is optimized for each class. The encoder is based on the recently proposed deterministic geometrically invariant scattering transform a.k.a ScatNet [28]. On the other side, the decoders are trained independently for each class to ensure the best class reconstruction accuracy.

We use the IB formulation as the theoretical basis for the fully distributed system. At the same time, the mechanism of exact information compression in the IB is not fully understood and there are various interpretations how the deep network tries to compress information by keeping the only most important information in the latent representation for the targeted classification task. The original work [6] suggests that the stochastic gradient descent places the noise to the irrelevant dimensions of the latent space at the second stage of training. Other authors [31] interpret the IB compression as clustering, where several inputs are clustered together, if they contain the same information according to the assigned class labels. Otherwise, VAE [32] and Adversarial Auto-Encoders (AAE) [20] try to produce the latent space that follows some pre-defined distributions, where the IB *compression* can be controlled by a proper selection of the dimension of the latent space or addition of noise to some dimensions or shaping the distribution of the latent space by an introduced prior.

In this work, we proceed with a hypothesis that the IB is achieved by the direct compression of certain dimensions in the latent space representation, even when the dimension of latent space is larger than the input one. At the same time, the selection of dimensions or groups of dimensions referred to as channels in the latent space to be compressed is based on the analysis of class common features. The lack of the knowledge of common dimensions in the considered formulation of distributed classification between the classes is compensated by the known properties of the scattering transform [33], obtained with ScatNet [28]. The low frequency channels of ScatNet represent low resolution data that are very correlated for all classes. Therefore, its lossy representation corresponds to the selective compression suggested by the IB principle.

## 5. Implementation Details

### 5.1. Training of Local Encoders

In this paper, we proceed with the local compressing encoders $E_{\phi_{m}}\left(\cdot \right)$ consisting of a deterministic feature extractor f(·), followed by a learnable compressor $Q_{\phi_{m}}\left(\cdot \right)$: $E_{\phi_{m}}\left(\cdot \right)=Q_{\phi_{m}}\left(f\left(\cdot \right)\right)$. The compressing encoding minimizes $I_{\phi_{m}}\left(X;Z\right)$ for the classifying purposes. In our setup, the feature extractor $f\left(\cdot \right)=S^{J}\left(\cdot \right)$ is fixed to be the scattering transform of deepness *J* for all classes as defined in [28]. There are several reasons for this choice: (i) the scattering transform is known to preserve the energy in the Fourier domain [34], and is highly sparse and invariant to some geometrical transformations [33], i.e., it produces the same latent space representation f(X) for small variability in X, (ii) in turn it needs less training examples to ensure invariance to geometrical transformations as shown in [28], where the authors show that the ScatNet of depth 2 with a simple linear SVM can achieve better classification accuracy for the smaller amount of training samples and (iii) the invariance and sparsity of the latent representations also help better training the decoding due to smaller variability and simpler (sparse) manifold, but also (iv) the invariant and deterministic scattering feature extraction brings interpretability of the latent representation to choose the compression strategy for unseen classes. The last is very important for the considered distributed setup, where no information about the classes is shared between the nodes.

In following, we consider the details of implementation of the fixed and class-independent scattering transform f(.) and learnable quantizer $Q_{\phi m}\left(.\right)$.

#### 5.1.1. Structure of the Scattering Transform

The feature extractor used to encode X is a deep scattering convolutional network defined in [28] of depth *J* equal to 2 or 3: $f\left(\cdot \right)=S^{J}\left(\cdot \right)$. We recall from Section 4 that the role of the feature extractor f(·) is to provide an exhaustive and qualitative description of X in such a way that the subsequent compression can select only the strictly relative components for the classification towards M. This role falls perfectly to the scattering transform $S^{J}\left(\cdot \right)$, which can produce on demand more or less features of X according to its depth *J*. If some data need very fine features to separate between classes by the compression, a deeper decomposition of $S^{J}\left(\cdot \right)$ is required. Table 1 presents the number of features extracted by $S^{J}\left(\cdot \right)$ according to the depth *J*, and the way in which these features are obtained.

The scattering extraction defined in [28] involves using a wavelet [34] basis $\psi_{j}^{\alpha }\left(u\right)=2^{-2j}\psi \left(r_{-\alpha }2^{-j}u\right)$, where ψ is the Morlet mother wavelet, 1≤j≤J is the scale and $r_{-\alpha }$ is the rotation by −α with $\frac{\alpha }{2\pi }\in Z/LZ$ the finite group of *L* elements. It also involves the use of the absolute function as an activation function applied after convolutions with the wavelets and a local averaging $\Phi_{J}$ on a spatial window of scale $2^{J}$. Each feature channel is of size $H/2^{J}\times W/2^{J}$, where H×W is the original image size. Table 1 shows the dimension of the scattering representation according to *J* and the initial size of a realization x of the random variable X.

**Figure 6:**
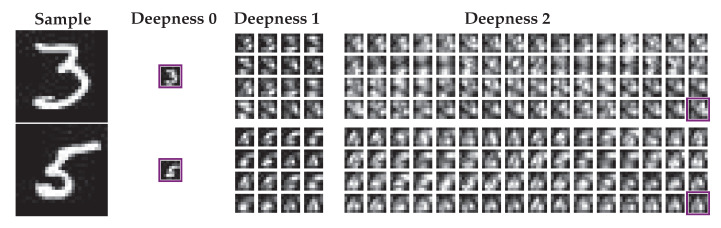
Scattering representation, feature selection and compression used for the bottleneck of our experimental setup. This figure shows the encoded representation for two MNIST samples, with the scattering transformation of deepness J=2. The feature selection is represented by the violet frames at scattering deepnesses 0 and 2, which selects only the two extreme channels of these representations. These two steps are deterministic and identical for each node. The second step of compression consists of quantizing the channel of deepness 0 with a node-dependent dictionary as shown in Figure A2.

**Figure A1:**
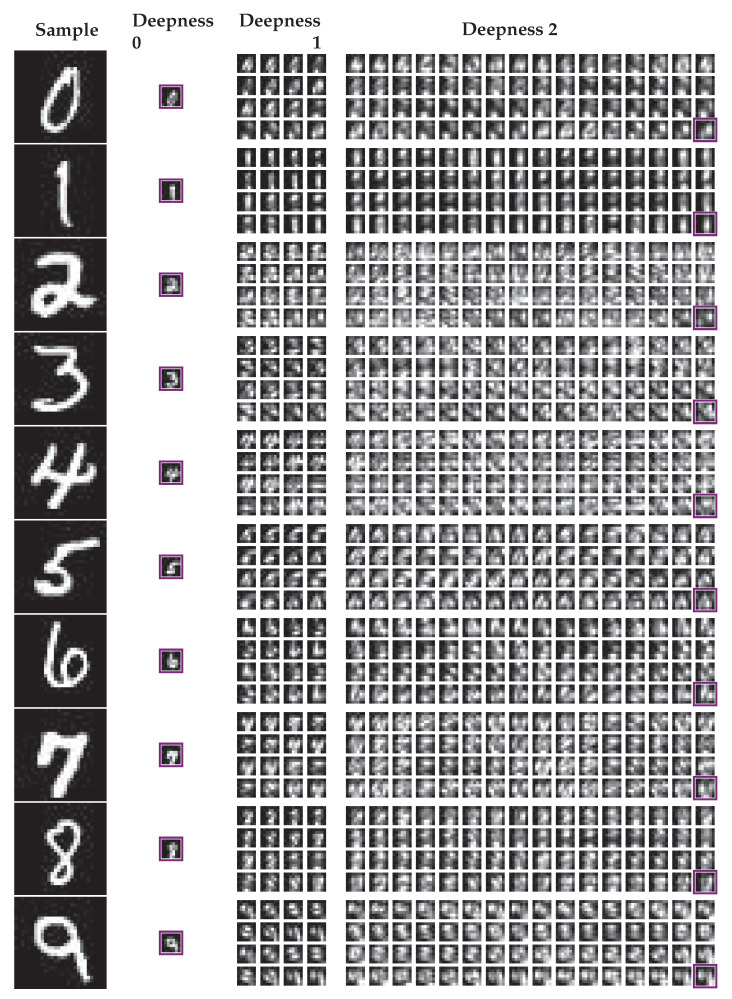
For 10 MNIST samples: (1) their encoded representation, which is a scattering transformation of deepness *J* = 2 and (2) the first step of compression used for the bottleneck of our experimental setup by the selection of only the two extreme channels inside violet frames. These two steps are deterministic and identical for each node. The second step of compression consists of quantizing the channel of deepness 0 with one of the dictionaries described in Figure A2, depending on the node where the process occurs.

The interpretation of the scattering feature space helps us choose the compression strategy for our experiments. As described in Table 1, the size of $S^{J}\left(x\right)$ is $H/4\times W/4\times N_{S^{J}}$ (with the format Height×Width×Channel) when x is a grayscale input image of size H×W; and when x is a color input image of size H×W×3 the size of $S^{J}\left(x\right)$ is $H/4\times W/4\times 3N_{S^{J}}$. Each channel of deepness δ≤J of the scattering transform $S^{J}\left(x\right)$ corresponds to a fixed parameter path $\alpha_{1},\cdots ,\alpha_{\delta }$, and $j_{1}<\cdots <j_{\delta }$ applied to the input image. The channels are ordered by increasing depths δ<J and parameters ${\left\{\alpha_{d},j_{d}\right\}}_{d=1}^{\delta }$ of their corresponding path, therefore the first channel $S_{0}^{J}$ is only a blurry version of x. For a better visualization and understanding, we give examples of the 81 channels obtained by the scattering transformation of two MNIST samples in Figure 6 and more examples are shown in Figure A1.

#### 5.1.2. Training of Local Quantizers

As shown in Figure 4, because the local encoding-decoding node was trained on the distribution of a class *m* its rate-distortion curve (RDC) will be sub-optimal for the distribution of another class $m^{\prime }$ and it will be above the RDC for the distribution of the class *m*, as soon as the distribution of these classes do not overlap in the considered space. Consequently, we target a rate $R_{Q}$ for the local node encoder–decoder, where the RDC for the dedicated class distribution is highly separable from the RDCs of other class distributions. For a sake of simplicity and interpretability, we have selected the same compression strategy and the same rate for all nodes.

The compression strategy is hybrid: (i) we want to quantize the channels with the lowest entropy, e.g. the channels that produce the same output for the in-class samples, and (ii) keep the channels with the lowest inter-class mutual information. The interpretations of the scattering transform channels given in Section 5.1.1 allows us to make the choice of: (i) quantizing the first channel $S_{0}^{J}$, (ii) keeping as is the channels of index larger than a given $i^{⋆}$, and (iii) suppressing all channels $S_{2}^{J},\cdots ,S_{i^{⋆}-1}^{J}$. In the local node for the class *m*, the encoding $z_{m}\left(x\right)=E_{\phi_{m}}\left(x\right)=Q_{\phi_{m}}\left(S^{J}\left(x\right)\right)$ of a given sample x is defined by:

(12) $$z_{m}^{\left(1\right)}\left(x\right)=\underset{q\in Q_{m}}{arg min}{\langle S_{0}^{J}\left(x\right),q\rangle }_{CS},\;z_{m}^{\left(2,\cdots ,N_{z}\right)}\left(x\right)=S_{i^{⋆},\cdots ,N_{S^{J}}}^{J}\left(x\right),$$

where $N_{z}=N_{S^{J}}+2-i^{⋆}$ is the number of channels of z, ${\langle \cdot ,\cdot \rangle }_{CS}$ is the cosine similarity and $Q_{m}$ is the codebook of centroids for the given class *m* used for the vector quantization of $S_{0}^{J}\left(x\right)$. For our experiment, it is made up of the centroids $q\in Q_{m}$ of a K-means pre-trained on ${\left\{S_{0}^{J}\left(x\right)\right\}}_{x\in D_{m}}$, i.e., the training samples coming from the first scattering channel of the local data class *m*. The quantized and kept channels are highlighted with violet frames for some MNIST samples in Figure 6 and Figure A1.

### 5.2. Training of Local Decoders

The architecture of the decoder $D_{\theta_{m}}$ ($1\leq m\leq N_{m}$) of class *m* is presented in Table 2. As suggested by Equation (10), its parameters $\theta_{m}$ are trained and optimized only over the dedicated class dataset $D_{m}$ by minimizing the distortion between the original and locally reconstructed samples. The distortion measure used in this experiment is the $\ell_{1}$ loss, which has been proven to generate finer images than $\ell_{2}$ loss [35]. One could also go further and train it jointly with the adversarial loss like in [7], but the simple use of $\ell_{1}$ is shown to produce satisfactory results on our experiments with lower complexity. We use the Adam optimizer [36] to find:

(13) $$\begin{array}{c} {\hat{\theta }}_{m}=\underset{\theta_{m}}{arg min}E_{p_{D_{m}}\left(x\right)}\left[∥{\hat{x}}_{m}{-x∥}_{1}\right],\;\mathrm{with}\;{\hat{x}}_{m}=D_{\theta_{m}}\left(E_{\phi_{m}}\left(x\right)\right), \end{array}$$

where $\phi_{m}$ corresponds to the parameters of encoding considered in Section 5.1, namely the codebook $Q_{m}$, the index $i^{⋆}$ and the scattering depth *J*.

It is important to point out that different classes might have different complexity of manifolds. To balance the same reconstruction error for different local encoder–decoder pairs, we assumed that the reconstruction error for all nodes should be approximately the same. Given different complexity of data manifolds for various classes, it can be achieved either by optimizing the structure of encoder–decoder pairs or adapting the number of epochs per each node. In this work, we proceeded with later and kept the structure of encoder–decoder fixed for all classes and just adapted the number of epochs to ensure approximately the same reconstruction error at the training stage.

### 5.3. Central Classification Procedure

**Figure 7:**
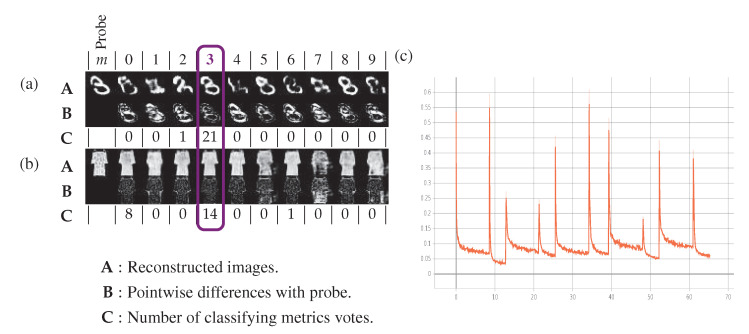
(**a**,**b**): Examples of reconstructions and classification on class 3 samples from (**a**,**b**) FashionMNIST dataset. The first column is for the probe and the following columns are for the results of the local nodes. The first row is for the names of the local nodes, rows A are for the probes and their reconstructions, rows B are for spatial errors, whereas rows C count the number votes for the corresponding node label given by the experimented classifying metrics among $d_{\ell_{1}}$, $d_{VGG}$ and ${\left\{d_{t}\right\}}_{t}$. One distance is incorrect in (**a**): $d_{0}$ vote for m=2. Nine distances are incorrect in (**b**): $d_{0},d_{.13},\cdots ,d_{.19}$ vote for m=0 and $d_{.9}$ vote for m=6. (**c**) TensorBoard of the converging training of the 10 local nodes.

Given a probe x coming from the testing dataset $D_{\mathrm{test}}$, we pass it through the $N_{m}$ class-dependant local node encoder-decoders and communicate the $N_{m}$ reconstruction errors $\left(e_{1},\cdots ,e_{N_{m}}\right)$ to the central node. As shown in Figure 4 and Equation (11), the probe x is classified according to minimum of the reconstruction error: $\hat{m}={arg min}_{1\leq m\leq N_{m}}e_{m},\;\mathrm{where}\;e_{m}=d\left({\hat{x}}_{m},x\right)$. The spatial differences between the probe and its reconstructions contributing to these errors are shown in the third and sixth lines of Figure 7 to exemplify the underlying process. We tested different classifying losses than the training $\ell_{1}$ loss. Experimental metrics for the distortion measurements considered in this paper include the following reconstruction errors: ${\left\{e_{m}\right\}}_{m=1}^{Nm}$:

- the Manhattan distance $d_{\ell_{1}}$,
- the perceptual distance $d_{VGG}$ defined in [37],
- the pseudo-distance $d_{t}$, which counts the number of pixels with an absolute error larger than a threshold *t*:
  (14) $$\begin{array}{c} \begin{array}{cc} d_{t}\left(\hat{x},x\right)=\sum_{i=1}^{N_{x}}{𝟙}_{\left|\hat{x}\left[i\right]-x\left[i\right]\right|\geq t},\;\mathrm{where}\;{𝟙}_{\left|\hat{x}\left[i\right]-x\left[i\right]\right|\geq t} & =\left\{\begin{array}{cc} & 1,\;\mathrm{if}\;\left|\hat{x}\left[i\right]-x\left[i\right]\right|\geq t,\\ & 0,\;\mathrm{else}. \end{array}\right. \end{array} \end{array}$$

For too small or large thresholds *t*, the pseudo-distance $d_{t}\left(.,.\right)$ fails to really capture reconstruction errors. For instance, for any images $x_{1}$ and $x_{2}$ of the same size $N_{x_{1}}$ with pixel values ranging from 0 to 1, $d_{0}\left(x_{1},x_{2}\right)=N_{x_{1}}=N_{x_{2}}$ and $d_{2}\left(x_{1},x_{2}\right)=0$. For this reason, Section 6.1 presents the classifying results experimented with $d_{t}$ for 6 different median value thresholds: 0.2,⋯,0.7.

## 6. Experiments

The experimental validation is performed with untrained encoding and controlled compression. To investigate the importance of compression at the encoding step, we considered the encoder presented in Figure 1 and Figure 4, consisting of feature extractor f(·) followed by a controlled compression $Q_{\phi_{m}}\left(\cdot \right)$ of these features. The decoders $D_{\theta_{m}}\left(\cdot \right)$ are trained for each class and corresponding nodes. Our untrained feature extraction f(·) is performed by the scattering transform defined in [33], gaining in invariance to geometrical transformations [28] and facilitating the learning of neural networks with this sparse representation as an input [5]. This experimentation validates the theoretical approach to challenging datasets for decentralized classification, even though it is a simple task for centralized ones.

### 6.1. Results

MNIST [15] and FashionMNIST [38] are fairly simple tasks for the common centralized supervised deep classifiers, but it is not the case for decentralized models, where it is challenging to learn fine-tuned decision boundaries, when restricted gradients are communicated to the central node. As mentioned in the previous sections, the aim of the experiment is to present the results that practically confirm the theory discussed in Section 4. The compression parameters have been fixed beforehand to simplify the learning and depend on the used dataset.

Our results in terms of classification error: (1) for MNIST are provided in Table 3 for which we achieve the state-of-the-art results with exactly 0 error on the testing dataset, and (2) for FashionMNIST are provided in Table 4 for which we have competitive results with centralized classifiers. The encoding parameters *J* (scattering deepness), *K* (number of centroids), $i^{⋆}$ (first kept channel in compression) are different between both datasets because they have different statistics and Fourier spectra reflected in the scattering transform. Our results considerably outperform the Federated Averaging for Non-IID-FL setup, and the perfect MNIST classification is not a fluke. In Table 3, the error on the training dataset is 0% for half of cases of classifying metrics, and only the $d_{.4}$ classifying loss gives 0% of error on the testing dataset for 3 cross-validation sessions. To compare with FL, we present the results for Federated Averaging from [13], where it is shown that from IID-FL to Non-IID-FL, there is a drop in performance in the classification accuracy from −3% to −74% depending the model and data used, and the distribution of the data across the local nodes.

#### 6.1.1. MNIST

We use the following parameters: J=2, K=5, $i^{⋆}=81$ (only the last subband is kept), batch size is 128 and the learning rate is $10^{-5}$. This implies that the size of the scattering features has a dividing factor $2^{J}=4$ from the MNIST original image size 28×28. The compression of the scattering representation goes from a 81×7×7 tensor (channels first) to a 2×7×7 with the first channel quantized by a codebook of K=5 centroids. The compression rate of the feature vector is $80\log_{2}\left(5\right):1$. The training of the 10 class-dedicated encoder-decoders described in Section 5.2 is performed with the Adam [36] optimizer. For each local node, their dedicated training dataset $D_{m}$ is sampled in their entirety at each epoch and the 10 local training losses are shown in Figure 7c: the training is very stable and converges. The structure of the decoder is fully convolutional and described in Table 2 with J=2: the size of input is 2×7×7 (channel first) followed by a sequence of 7 layers alternating 4 convolutions and 3 batch-normalizations [45], ReLU activations [46], and tanh activation for the output layer.

#### 6.1.2. FashionMNIST

We used J=2, K=5, $i^{⋆}=18$: only the paths of deepness 2 (for more details see Table 1) are kept, otherwise the reconstructions have too large distortions, batch size is 128 and the learning rate is $10^{-5}$. If we keep the same $i^{⋆}=81$ as for MNIST, the reconstructions have too large distortions. This compression does not hold enough information for optimal reconstruction. With $i^{⋆}=18$, the compression of the scattering representation goes from a 81×7×7 tensor (channels first) to a 65×7×7 with the first channel quantized by a codebook of K=5 centroids. The compression rate of the feature vector is $\frac{5\log_{2}\left(5\right)}{4}:1$. Under these settings, the 10 independent class-dedicated encoder–decoder training converges with the same behavior as in Figure 7c. Nevertheless, the classification accuracy shown in Table 4 is less than for MNIST. This is due to the fact that when the compression rate is too small, the class-dedicated encoder-decoders are less separable as shown in Figure 4. Also, playing with the rate $R_{Q}$ and augment from K=5 to K=15 the length of the 10 local quantizing codebooks $Q_{m}$, the classification accuracy drops from 89.9 to 82.81, hence confirming the rate-distortion theory interpretation. The structure of the decoder is a fully convolutional and described in Table 2 with J=2: the size of input is 65×7×7 (channel first) followed by a sequence of 7 layers alternating 4 convolutions and 3 batch-normalizations [45], ReLU activations [46], and tanh activation for the output layer.

## 7. Discussion

### 7.1. Investigation of the Bottleneck Role

**Figure 8:**
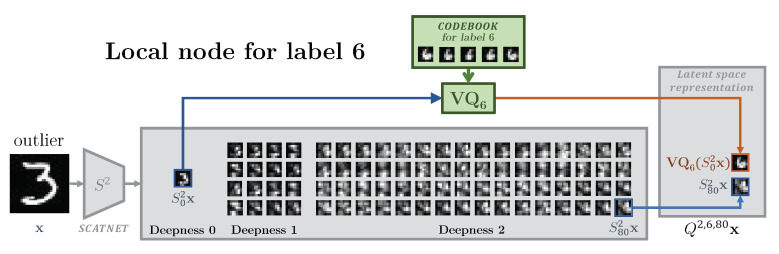
Detailed presentation of the different steps of compression for the local node 6, defined in Section 5.1.2, from the scattering representation of an outlier with label 3 to its compressed representation.

**Figure 9:**
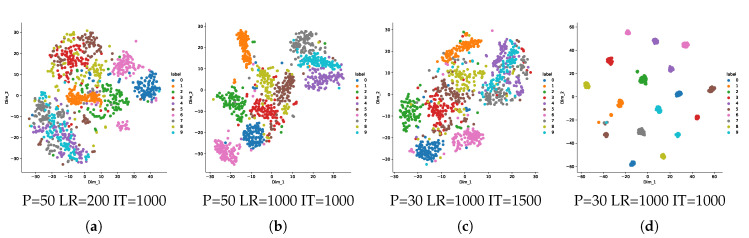
tSNEs showing representations of the MNIST data manifolds at different steps of the classifying process: (**a**) for the raw data, (**b**) for their scattering representations as output of ScatNet with all deepnesses and coefficients shown in Figure 8, (**c**) after suppressing the 79 intermediate channels, when only the two blue framed channels of the scattering representation are kept as shown in Figure 8, and (**d**) after quantization of the first channel by node quantizers of the same class as the samples as shown in the latent space representation of Figure 8, and whose dictionaries are shown in Figure A2. P, LR and IT respectively stand for the perplexity, the learning rate and the number of iterations of the tSNEs.

To investigate and experimentally justify the assumptions behind the bottleneck compression described in Section 5.1.2, we describe the steps of compression in Figure 8 and show the corresponding representations of data manifolds at these different steps of compression in Figure 9: the over-complete sparse and geometrically invariant scattering transform representations shown in (b) already give a higher separability than the raw data of (a). The subband selection (c) and quantization (d) proposed in Section 5.1.2 increase separability between the classes. We highlight that the tSNE shown in (d) is assuming an ideal quantization, where the scattering transform channel of deepness 0 is assigned to the image taken in the corresponding label dictionary; in reality, the quantization is done on each class node with their local dictionary.

### 7.2. One-Class Manifold Learning for Separability

**Figure 10:**
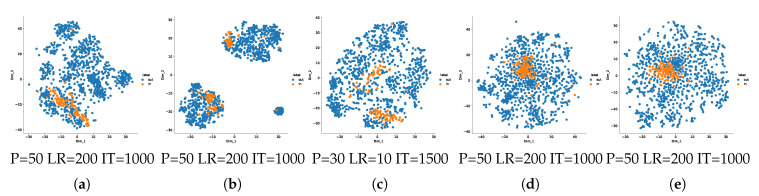
tSNEs showing representations of inliers and outliers on MNIST data manifolds for the node of label 9, at different steps of the encoding-decoding process. inliers are samples of label 9 and outliers are samples of the rest of labels: (**a**) for the raw data, (**b**) for the data in node 9 after ScatNet and compression as described in Section 5.1.2, (**c**) for the data in node 9 after reconstruction by the decoder, (**d**) for the error of reconstruction with the original samples as shown in raw B of Figure 7a, and (**e**) for error of reconstruction after application of the optimal thresholding with t=0.4 presented in Section 5.3 and Table 3. More results for all nodes are shown in Figure A3, Figure A4 and Figure A5. P, LR and IT respectively stand for the perplexity, the learning rate and the number of iterations of the tSNEs.

An ideal case for the ON–OC–IBC would be to have ideal anomaly detectors or one-class classifiers at each node. This prompts us to investigate the one-class separating power of each local node. We experimentally show this with tSNEs in Figure 10 for node 9 and Figure A3, Figure A4 and Figure A5 for the others. After the compression in the bottleneck, the inliers and outliers tend to separate but in different subgroups, whereas after the reconstruction, the manifold of inliers seems to be a single nested set, separated from the outliers. At the end, we see that the reconstruction error followed by the non-linearity $d_{.4}$ applied to each difference plays an important role for the final classification at the central node, this is made evident from the improved separability of in- and outlier manifolds in the t-SNE representations.

### 7.3. Influence of Feature Selection and Link to the Rate of Compression

**Figure 11:**
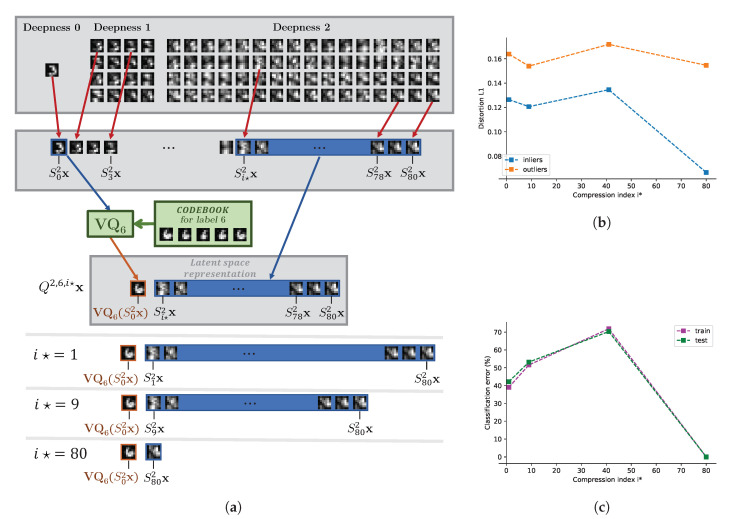
(**a**) presents how different rates of compression are achieved in order to obtain the rate-distortion curves (**b**) for the reconstructions at node 7, and (**c**) for the accuracies of classification at the central node. In (**a**) different rates are achieved by changing the index $i^{⋆}$ defined in Section 5.1.2 before which the scattering channels are suppressed during compression. In (**b**), the distortion on the y-axis is measured by the $\ell_{1}$ norm of the reconstruction error, and the rate on the x-axis is represented by the value of $i^{⋆}$, the scattering channel index from which the information is kept at the compression stage, as described in Section 5.1.2; the greater $i^{⋆}$ is, the more compression occurs. The blue curve is for inliers (samples of label 7 used to train the node) and the orange curve is for outliers. In (**c**) the classification accuracies are given on the training and testing dataset for different rates represented by $i^{⋆}$. The number of centroids for the “0”-subband of ScatNet was fixed to K=5. (**a**) Index $i^{⋆}$ and rate of compression; (**b**) Rate-distortion curve for the local node of label 7 on MNIST data; (**c**) Classification error (the lower - the better) on MNIST data for the central node.

Figure 11a for the node of label 7 and Figure A4 for all other nodes gives an experimental proof of the rate-distortion concept for classification on a fully decentralized systems presented in Figure 4. According to the features produced by the ScatNet, the compression can be controlled by two parameters to get the best separability between the in- and outliers rate-distortion curves: (1) for better classification at the central node, several channels from the scattering transform is chosen with the parameter $i^{⋆}$ defined in Section 5.1.2, when J=2, the scattering transform has 81 channels, in consequence, when $i^{⋆}=80$, only two channels are kept, the first and the last one; (2) and the second parameter of compression is *K*, the number of elements in the codebook used for the quantization of the first channel.

#### 7.3.1. Influence of the Parameter $i^{⋆}$

**Figure A2:**
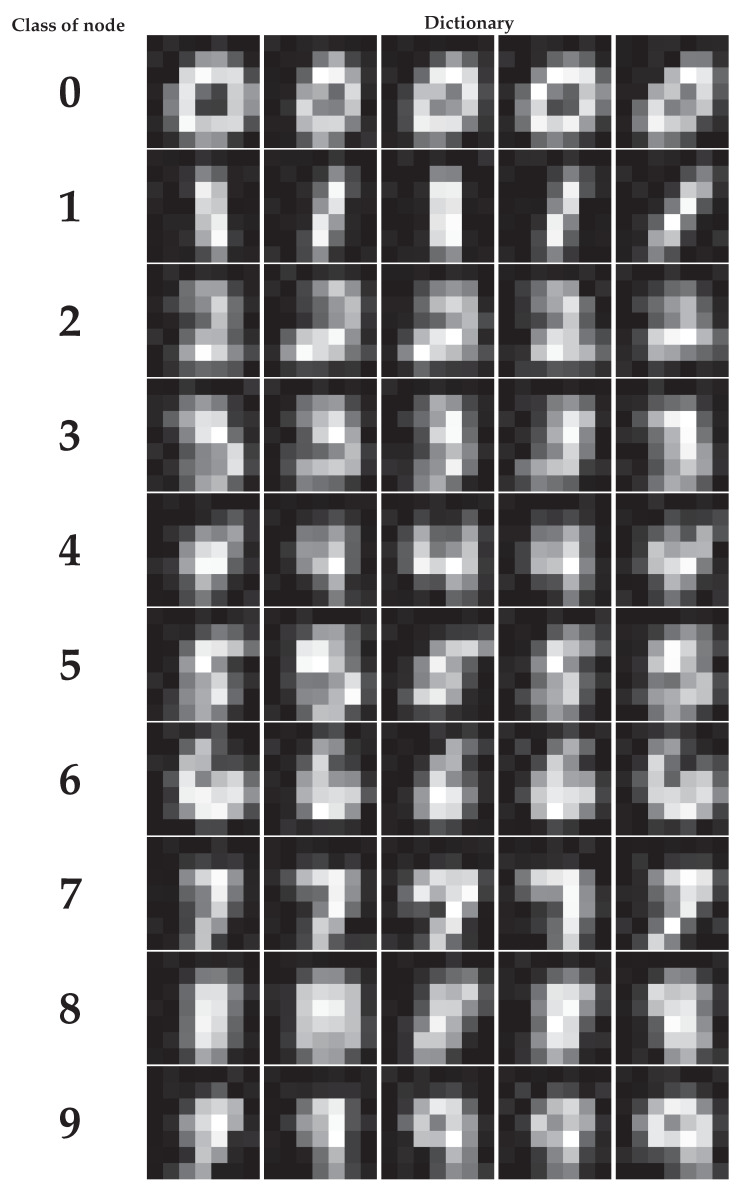
MNIST dictionaries for nodes used for the quantization of the first channel of the scattering transform. This first channel is a reduced version of the image obtained by a Gaussian blurring. The dictionary for one node consists of the centroids resulting from K-means applied to the training data of a node, with *K* = 5.

**Figure A3:**
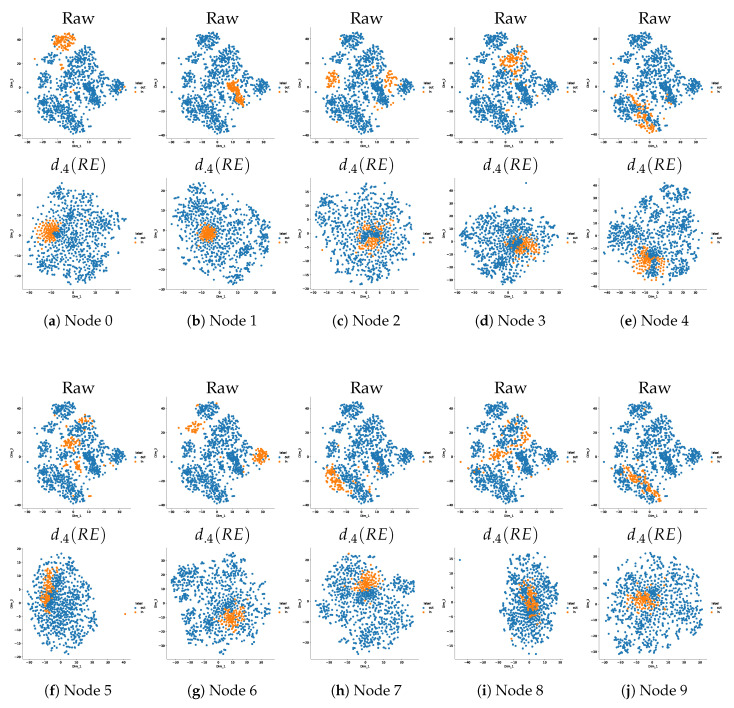
MNIST manifolds description by tSNE in terms of in- and outliers for raw data and reconstructed errors RE with the classifying non-linearity $d_{.4}$. Figure 7 shows that inliers have visually quite good reconstructions whereas outliers reconstructions are visually different from their raw version. This visual separability is confirmed by the modified in- and outliers manifolds for each node. For the raw data, the parameters are perplexity P = 50, learning rate LR = 200 and number of iterations IT = 1000. For the reconstructed, parameters are given in the detailed Figure A4 and Figure A5.

**Figure A4:**
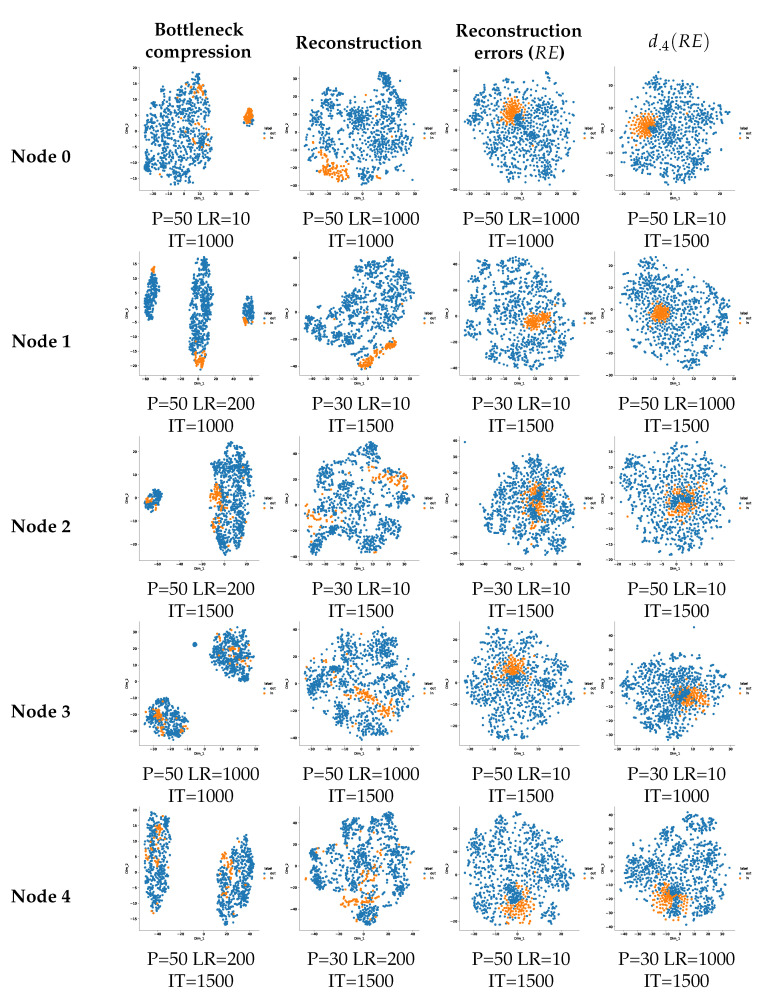
Manifold learning of our setup: for each node from label 0 to label 4, tSNEs show the manifolds for MNIST data at different stages of the proposed local auto-encoders. In orange are inliers samples (local data used for training, of the same class as the node label) and in blue are outliers samples(data unseen during training which originates from a different class). First column: for the output of the bottleneck; second column: for the reconstructed samples at the output of the decoders; third column: for the reconstruction errors with the original samples; and last column: for the reconstruction errors after application of the non-linearity metric $d_{._{4}}$ which classifies best at the central node. P stands for perplexity, LR for learning rate, and IT is the number of iterations. We can see that each step plays a role for the local manifold learning and separation power with outliers.

**Figure A5:**
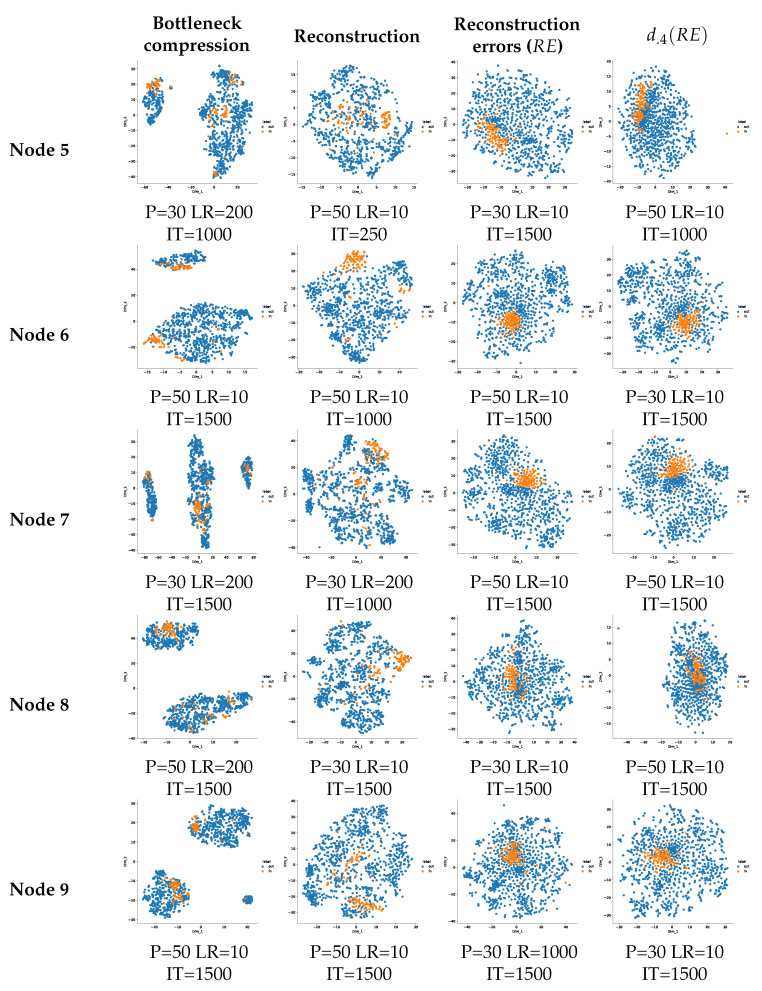
Manifold learning of our setup: for each node from label 5 to label 9, tSNEs show the manifolds for MNIST data at different stages of the proposed local auto-encoders. In orange are inliers samples (local data used for training, of the same class as the node label) and in blue are outliers samples(data unseen during training which originates from a different class). First column: for the output of the bottleneck; second column: for the reconstructed samples at the output of the decoders; third column: for the reconstruction errors with the original samples; and last column: for the reconstruction errors after application of the non-linearity metric $d_{.4}$ which classifies best at the central node. P stands for perplexity, LR for learning rate, and IT is the number of iterations. We can see that each step plays a role for the local manifold learning and separation power with outliers.

The link between the rate-distortion separability in the local nodes and the classification accuracy in the central node is confirmed by the rate-distortion curves of Figure 11b, where the highest accuracy of classification is achieved when $i^{⋆}=80$, which means a local quantization of the scattering transform channel 0 by the dictionaries of Figure A2, stacked with the last channel of index 80 and a suppression of all the intermediate scattering channels. With less compression, when $i^{⋆}$ is smaller, the in- and outliers are less separable in the rate-distortion curves. It should be pointed out that we interpret the rate of compression as several selected channels. We did not investigate which sub-bands out of 80 are the most distinguishable due to high complexity and simply controlled the number of sub-bands indexed in the descending order. Obviously, these parameters can be optimized to further increase the accuracy of classification.

#### 7.3.2. Influence of the Parameter *K*

Table 5 summarizes for MNIST dataset how the classification error on the central classifying node is impacted by the size *K* of the codebook for the quantization of the first scattering channel. We fixed $i^{⋆}=80$ for this experiment for classification purposes and used the classifying metric $d_{.4}$. This experiment shows that:

- K=5 achieves smallest classification error in the central node,
- near K=5 there is a smooth behavior and K=5 remains optimal in terms of classification.
- K=1 leads to the overfitting as the table shows a drop of performance between the train and the test datasets,
- for K>5, the table shows a drop in performance due to non-separability of rates of distortions between nodes.

It is important to note that for apart from K∈{4,5,6}, no search for the best hyperparameters were performed. This is an important factor as for K=5, the central classification starts to perform very well given that that same reconstruction accuracy is achieved for all nodes with enough epochs. However, if nodes are trained with different errors of reconstruction, this imbalances the whole system and leads to erroneous classification at the central node. We assumed that a good way to measure the quality of learning of one node is to use its training loss curve across the time shown in Figure 7c: we fixed each nodes to stop learning after their training loss reach 0.065, with a maximum variation of 10% during 10 epochs. The first thresholding criteria ensures that all nodes have similar distortion measures, and the second criteria ensures that all nodes learned quite well their own class manifold. We also added a maximum number of epochs for practical reasons, and for the results given in Table 5, apart from K∈{4,5,6}, it is this last criteria which stopped the trainings.

It is also important to note that we use the $\ell_{1}$ norm to estimate the reconstruction error at training, but for the recognition/testing we use the considered $d_{.4}$ metric. This is a potential source of the observed performance but due to the non-differentiability of these metrics we do not consider them in the training loss.

The hyperparameter search, including *K*, $i^{⋆}$ and the stopping criteria, remains an open question for us that we would like to answer in future studies. We also have in mind to make the rate of quantization learnable, but this is not under the scope of this paper.

## 8. Conclusions

The relative competitive results presented in Table 3 and Table 4 constitute a proof of concept for our fully decentralized model. We want to emphasize that it is constructed from the interplay between *information bottleneck* principles and recent attempts to make machine learning architectures simpler and more interpretable (see [28,33,47]).

**Shannon’s Rate-Distortion theory and IB principles:** The main novelty is that we introduce a compression by partially suppressing and quantizing information in the latent representations of untrained feature extractors. We introduce higher reconstruction errors for more separability of the classes like in Figure 4. We demonstrate that a central node which does not share any information about classes for training and classification, can achieve competitive classification performance in comparison to classical systems.

**Compression principle:** Following the IB principles for two close classes, one should learn only what makes these classes unique, and compress common data in the latent representation. Scatnet provides universality and interpretability of its representations. We only quantize the first channel corresponding to a blurred image of the probe. This is the most common component of the dataset. Thus, we suppress much information in the first channels while retaining the last channels which hold the high frequency information of the probe and are unique for each class. This introduces more separability in the learned manifolds. Nevertheless, we should keep enough information to reconstruct accurately for the inliers.

**Choice of parameters*J* and $i^{⋆}$:** For simple datasets like MNIST, we can suppress many ScatNet channels, and still retain enough information to accurately reconstruct the inliers. For a more complex dataset, we should suppress less information ($i^{⋆}$ smaller). With more scattering features (*J* larger), one can maintain separability. A trade-off expressed in Equation (10) is made between the rate and the distortion, and could also be optimized to learn these parameters. One can use a recent framework [48] to estimate mutual information between the channels of ScatNet to choose which channels contain common information to be suppressed by quantization. This will be our future line of research for more complex datasets.

**Back to “matched filtering” based on auto-encoding**: We show that it is possible to reach and even outperform more classical centralized deep-learning architectures implemented in a federated, decentralized model. The most adopted interpretation of a deep-learning-based classification paradigm is that it can capture and accurately approximate the decision boundaries between the classes in the multi-dimensional space. In return, it requires having all data in a common place to learn these boundaries. The state-of-the-art distributed deep-learning classification system mostly targets to optimize the rate of gradient exchange and potential leakages at the training stage in communication between the nodes and the centralized server. In contrast, we practically demonstrate that our classifier can be trained in a completely distributed way, when each node has access to data of its own class, gradients are not shared and other classes are unknown. Thus, the decision boundaries between the classes cannot be learned as such. In return, it suggests that we learn and encode a manifold of each class and only test the closeness of probe to this class at the testing stage. This conceptually link the proposed approach with the well-known in-signal processing concept of matched filtering.

**Data-management advantages**: Another consequence of the proposed framework is a possibility to decentralize the data to analyze and classify it. Such a method would allow the partition of work for analyzing data between different independent servers. Each pair of encoder–decoder might be independently trained with different training data, rendering big and maybe confidential data transfers unnecessary.

**Future work**: For the future research we aim at investigating the proposed framework on more complex datasets like, for example, ImageNet [49], Indoor Scene Recognition [50], Labeled Faces in the Wild [51]. The investigation of a robustness of the proposed framework against the adversarial attacks is an important open question for the future work as well as the studying of unbalanced decentralized systems where some classes could come from similar distributions or the situation where nodes could own different proportions of training data.

## Figures and Tables

**Table 1:** The number of growing scales paths until the deepness J=3. Each deepness parameters $j_{d},\alpha_{d}$ in a given path are parametrized by $0\leq \frac{\alpha }{2\pi }L<L$ for the rotations and $1\leq j_{d-1}<j_{d}<j_{d+1}\leq J$ for the scales. $N_{s^{J}}$ is the total number of scattering features channels given for deepness *J*, *H* is the height and *W* the width of x. These values are for gray-scaled images (×3 for RGB pictures).

Scattering Features for One Given	Number of	$S^{2}\left(x\right)$	$S^{3}\left(x\right)$	Tensors	$S^{2}\left(x\right)$	$S^{3}\left(x\right)$
Path by Growing Deepness	Channels	(J=2)	(J=3)	Sizes	(J=2)	(J=3)
$x⋆\phi_{J}\left(2^{J}u\right)$	1	1	1	$N_{S^{J}}$	81	729
$\left|x⋆\psi_{j_{1}}^{\alpha_{1}}\right|⋆\phi_{J}\left(2^{J}u\right)$	JL	16	24	Height	H/4	H/8
$\left|\left|x⋆\psi_{j_{1}}^{\alpha_{1}}\right|⋆\psi_{j_{2}}^{\alpha_{2}}\right|⋆\phi_{J}\left(2^{J}u\right)$	J2L2	64	192	Width	W/4	W/8
$\left|\left|\left|x⋆\psi_{j_{1}}^{\alpha_{1}}\right|⋆\psi_{j_{2}}^{\alpha_{2}}\right|⋆\psi_{j_{3}}^{\alpha_{3}}\right|⋆\phi_{J}\left(2^{J}u\right)$	J3L3	0	512			

**Table 2:** The decoder $D_{\theta }$ batch-normalizes and convolves the compressed scattering representation z; then it chains cycles of deconvolutions with batch-normalizations and ReLu activation functions until the probe size is recovered. The last activation function is the hyperbolic tangent. c=1 for gray-scaled images and c=3 for RGB images. The deepness *J* of the scattering encoding determines as well the deepness of the decoder.

Stage	Number of Channels	Filter Size	Stride	Size Scale	Activation
input $z_{m}=E_{\phi_{m}}\left(x\right)$	$N_{z}$			12J	
Batch Normalization					
Convolution	$2^{3(J+1)}c$	3×3	1×1		ReLU
Deconvolution	$2^{3J}c$	4×4	2×2	12J−1	
Batch Normalization					ReLU
Deconvolution	$2^{3(J-1)}c$	4×4	2×2	12J−2	
Batch Normalization					ReLU
⋮					
Deconvolution, output: $\hat{x}$	*c*	4×4	2×2	1	tanh

**Table 3:** MNIST classification error on the training and testing datasets for our *One Node–One Class–Information Bottleneck Classification* (ON–OC–IBC) setup with different classifying metrics, compared with the state-of-the-art centralized methods BMCNN+HC [39], EnsNet [40] and RMDL [41], which are based on merging sub-networks or aggregating their sub-predictions by majority voting, and the state-of-the-art Federated Averaging (FedAvg) on IID and Non-IID setup given in [12], where the IID setup corresponds to 10 nodes each with a uniform partition of the data of the 10 classes, and the Non-IID result is given with a similar setup as ours, with 10 local nodes and one class data per node, and differs to our setup by the fact that gradients are shared across local nodes.

	Centralized Methods						FedAvg	
Method	BMCNN + HC		EnsNet		RMDL		IID	Non-IID
Testing Data Error	0.16		0.16		0.18		1.43	7.77
	Proposed fully decentralized ON–OC–IBC							
Method	$d_{\ell_{1}}$	$d_{VGG}$	$d_{.2}$	$d_{.3}$	$d_{.4}$	$d_{.5}$	$d_{.6}$	$d_{.7}$
Training data error	1.5	0	3.1	1.5	0	0	0	1.5
Testing data error	4.6	3.1	1.5	3.1	0	4.6	6.2	7.8

**Table 4:** FashionMNIST classification error on testing dataset for the proposed ON–OC–IBC setup with different classifying metrics, compared with the state-of-the-art centralized methods such as RN18+FMix [42], which is a Mixed Sample Data Augmentation that uses binary masks obtained by applying a threshold to low frequency images sampled from Fourier space, and with classical CNN, CNN++ and LSTM described in [43], and the state-of-the-art Federated Learning methods such as FedAvg and WAFFLe [44], the Weight Anonymized Factorization for Federated Learning that combines the Indian Buffet Process with a shared dictionary of weight factors for neural networks. The results of these two methods are given for the Non-IID setup with only Z=2 data classes stored in each local nodes, either in a unimodal (Uni) way, with a 1:1 ratio of data present from both classes, or a multimodal (Multi) way, with a 1:5 ratio of data in each local node. For the ON–OC–IBC setup proposed, only Z=1 data class is stored in each local node, and there is no data distribution ratio, but the number of local nodes used is exactly the number of classes.

	Centralized Methods				FedAvg		WAFFLe	
Method	RN18+FMix	CNN	CNN++	LSTM	Uni	Multi	Uni	Multi
Testing data error	3.64	8.83	7.46	11.74	16.04	16.57	12.88	13.91
	Proposed fully decentralized ON–OC–IBC							
Method	$d_{\ell_{1}}$		$d_{VGG}$	$d_{.2}$	$d_{.3}$		$d_{.4}$	
Testing data error	10.1		12.2	12	13.1		14.4	

**Table 5:** Error of classification on the MNIST train and test datasets, for different values of the quantization parameter *K*, $i^{⋆}=80$ being set. We use the notation K=∞ when no quantization is performed on the first channel of the scattering latent representation. The classification metric is $d_{.4}$.

*K*	1	4	5	6	15	20	50	100	*∞*
on train (%)	80.4	19.1	0	19.0	89.6	91.1	91.8	91.1	90.2
on test (%)	90.2	23.9	0	24.4	89.7	91.2	92.1	91.2	90.2

**Figure A6:**
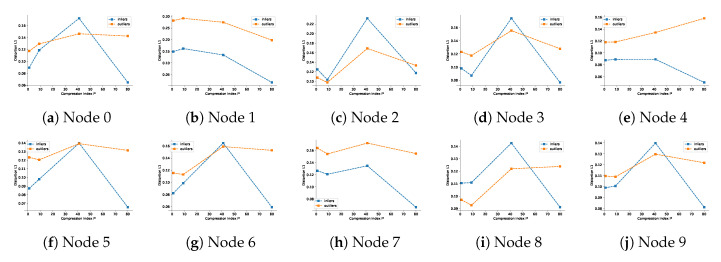
Rate-Distortion curves on MNIST distributions for each local node. The distortion on the y-axis is measured by the $\ell_{1}$ norm of the reconstruction error, and the rate on the x-axis is represented by the value of $i^{⋆}$, the scattering channel index from which the information is kept at the compression stage, as described in Section 5.1.2; the greater $i^{⋆}$ is, the more compression occurs. The blue curves are for inliers (samples with the same label as the local one used to train the node) and the orange curves are for outliers. For each node, there is a higher separability when $i^{*}=80$ which corresponds to the final setup presented in Section 6.1. These curves correspond to the theoretical ones presented in Figure 4 and $i^{*}=80$ corresponds to the optimal $R_{Q}$ for the best classification achieved. We note that for some nodes, when $i^{*}=41$, the distortion is larger for inliers than outliers, maybe the structure of the ScatNet representation is the cause. It also happens for node 8 when $i^{*}\in \left\{1,9,41\right\}$ which means that for these rates, outliers are better reconstructed by node 8 than samples of 8. This problem has also been commented in [21] when the classical outlier detector trained on the class 8 of MNIST perfectly reconstructs outliers. The authors in [21] bypass this problem with some structural tricks and regularization for the bottleneck of their model which actually increase the rate of compression. In our setup, when $i^{*}=80$, the node for label 8 reconstructs inliers better than outliers and can separate.

## Abbreviations

AE, Auto-Encoder; AAE, Adversarial Auto-Encoder; BMCNN + HC, Branching and Merging Convolutional Network with Homogeneous Filter Capsules; CNN(s), Convolutional Neural Network(s); CNN++, CNN with Batch Normalization and Residual Skip Connections; ELBO, Evidence Lower BOund; EnsNet, Ensemble learning in CNN augmented with fully connected sub-networks; FedAvg, Federated Averaging; FL, Federated Learning; GAN, Generative Adversarial Network; IB, Information Bottleneck; (Non-)IID, (Non-)independent identically distributed; IT, Number of iterations of tSNE; LR, Learning rate of tSNE; LSTM, Long-short term memory; MNIST, Mixed National Institute of Standards and Technology; MSE, Mean Squared Error; NN(s), Neural Network(s); ON–OC–IBC, One Node–One Class–Information bottleneck classification; P, Perplexity of tSNE; RMDL, Random Multimodal Deep Learning for Classification; RDC, Rate-Distortion Curve; ScatNet, Scattering Network; SKA, Square Kilometer Array; VAE, Variational Auto-Encoder. For all our diagrams, the colored trapezes represent NNs, and CNNs. The letters *E*, *D* and *C* are reserved for encoders, decoders and classifiers, respectively. The parameters of the networks are labeled with Greek indices. Columns represent vectors, with circles and squares being used for numerical and binary values, respectively.
